# The *SEP* homologous gene *TEMARY* regulates inflorescence phenotypes in *Hydrangea Macrophylla*

**DOI:** 10.1093/hr/uhae332

**Published:** 2024-11-26

**Authors:** Kenji Nashima, Tatsuya Uemachi, Kenta Shirasawa, Akifumi Shimizu, Toshiki Takeuchi, Tatsuya Obata, Sachiko Isobe, Mirai Azuma, Midori Akutsu, Yoshiko Nakazawa, Masaharu Kodama, Kiyoshi Namai, Takeshi Kurokura, Takuro Suyama

**Affiliations:** College of Bioresource Sciences, Nihon University, Kameino 1866, Fujisawa, Kanagawa 252-0880 Japan; School of Environmental Science, The University of Shiga Prefecture, Hassakacho 2500, Hikone, Shiga 522-0057 Japan; Department of Frontier Research and Development, Kazusa DNA Research Institute, Kazusa-Kamatari 2-6-7, Kisarazu, Chiba 292-0813 Japan; School of Environmental Science, The University of Shiga Prefecture, Hassakacho 2500, Hikone, Shiga 522-0057 Japan; School of Environmental Science, The University of Shiga Prefecture, Hassakacho 2500, Hikone, Shiga 522-0057 Japan; School of Environmental Science, The University of Shiga Prefecture, Hassakacho 2500, Hikone, Shiga 522-0057 Japan; Department of Frontier Research and Development, Kazusa DNA Research Institute, Kazusa-Kamatari 2-6-7, Kisarazu, Chiba 292-0813 Japan; College of Bioresource Sciences, Nihon University, Kameino 1866, Fujisawa, Kanagawa 252-0880 Japan; Tochigi Prefectural Agricultural Experiment Station, Kawarayacho 1080, Utsunomiya, Tochigi 320-0002 Japan; Tochigi Prefectural Agricultural Experiment Station, Kawarayacho 1080, Utsunomiya, Tochigi 320-0002 Japan; Tochigi Prefectural Agricultural Experiment Station, Kawarayacho 1080, Utsunomiya, Tochigi 320-0002 Japan; Tochigi Prefectural Agricultural Experiment Station, Kawarayacho 1080, Utsunomiya, Tochigi 320-0002 Japan; Faculty of Agriculture, Utsunomiya University, Mine 350, Utsunomiya, Tochigi 321-8505 Japan; Fukuoka Agriculture and Forestry Research Center, Yoshiki 587, Chikushino, Fukuoka 818-8549 Japan

## Abstract

Most *Hydrangea* species have inflorescences composed of two types of flowers: decorative flowers with showy sepals and plain nondecorative flowers. In wild plants of *Hydrangea macrophylla*, the decorative flowers are located at the periphery of the corymb, resulting in the lacecap phenotype. However, after the discovery of the mophead phenotype, in which decorative flowers are borne not only at the periphery but also on the entire inflorescence, it developed remarkably as a garden plant. In this study, we aimed to identify the gene controlling the inflorescence type and the mutations causing the mophead phenotype. Linkage analyses identified a *SEPALLATA* (*SEP)* homologous gene as a candidate gene, named *TEMARY*. We analyzed the genome sequences of *TEMARY* using several cultivars. The results revealed that the *H. macrophylla* cultivars had three types of loss-of-function alleles, and that the locus of the mophead cultivars consisted of only loss-of-function alleles. The phenotypes of 27 mophead cultivars could be explained by three types of loss-of-function *TEMARY* alleles. RNA-seq analysis and qRT-PCR analysis using two bud sport mutant lines related to the inflorescence type revealed that mophead mutants did not express *TEMARY* normally. These results suggest that *TEMARY* controls the inflorescence type and that mutations in this gene cause the mophead phenotype.

## Introduction

Most *Hydrangea* species have inflorescences composed of two types of flowers: decorative flowers with showy floral organs and plain nondecorative flowers. The sepals of decorative flowers are large and white, blue, pink, purple, and red, whereas those of nondecorative flowers are small and green. Decorative and nondecorative flowers of *Hydrangea macrophylla* differ in the shape of their sepals, number of floral organs, position of flowers on the inflorescence axes, and pedicel morphology [1, 2]. In contrast, the petal shapes are similar in both types of flowers, as are the stamens and pistils, which produce viable pollen and functional ovules, respectively. Most species in the genus *Hydrangea* bear corymb. In the corymb of *Hydrangea* species, the internodes on the main inflorescence axis are alternately short and long. In the corymbs of *H. macrophylla* and *H. serrata*, two decorative flowers are borne on each of the secondary inflorescences formed in pairs on the first and second nodes of the main inflorescence axis [3]. These four secondary inflorescences are located at the periphery of the corymb; therefore, the decorative flowers are also located at the periphery of the corymb, resulting in a frame-shaped phenotype. In the inflorescences of *H. macrophylla* and *H. serrata*, the frame-shaped phenotype is referred to as the lacecap phenotype. Furthermore, in these species, as in several other *Hydrangea* species, a mophead phenotype has been found, with many decorative flowers in the inflorescence. In the mophead phenotype, many decorative flowers with long pedicels completely overshadow nondecorative flowers with short pedicels, resulting in a globular inflorescence. This phenotype is found very rarely in wild populations of *H. macrophylla* or *H. serrata*; however, its discovery has led to the widespread use of these species as horticultural plants. Although this phenotype is a valuable trait in hydrangeas, the genes controlling it have not yet been elucidated.

The mophead phenotype of *H. macrophylla* is a recessive trait that appears when the mophead allele is homozygous or when different mophead alleles are combined [4, 5]. From the latter half of the 19th century to the beginning of the 20th century, three mophead cultivars of *H. macrophylla* from Japan and one from China were introduced into Europe and the USA [6]. Many mophead cultivars have been bred using these four germplasm lines and their descendants. Therefore, the number of mophead alleles utilized for breeding *H. macrophylla* is limited.

**Figure 1:**
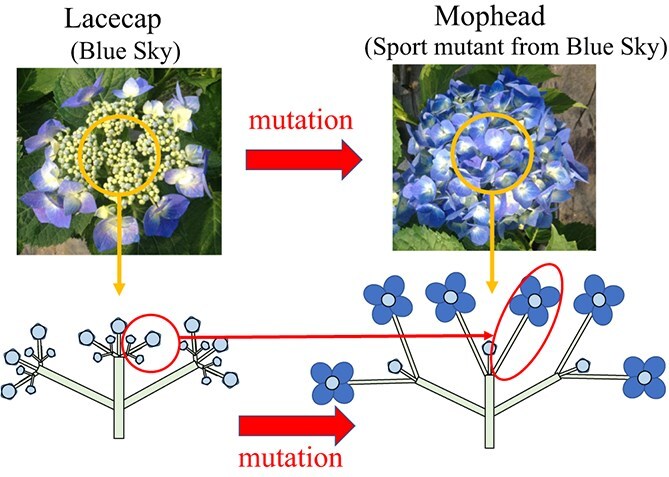
Changes in inflorescence structure associated with mutation from the lacecap phenotype to the mophead phenotype [2]. The mutation from the lacecap phenotype to the mophead phenotype is induced by the replacement of axillary higher order inflorescences with decorative flowers.

Uemachi et al. [2] compared the structure and developmental processes of the inflorescences of lacecap and mophead hydrangeas using a lacecap cultivar and mophead phenotype mutant (bud sport). The total number of flowers constituting the inflorescence decreased significantly because of the mutation from the lacecap to the mophead phenotype. The percentage of decorative flowers among all flowers in the inflorescence was 3.7% for lacecap and 66.5% for mophead. The fundamental characteristics of the mophead inflorescence structure were similar to those of the lacecap. In both phenotypes, nondecorative flowers were set on top of the inflorescence axes as terminal flowers, and pairs of axillary higher order inflorescences were set racemosely on most nodes of the axes. The only difference between the lacecap and mophead inflorescences was the structure of the upper nodes near the terminal flowers. Axillary higher order inflorescences were set on the upper nodes of the lacecap, whereas decorative flowers were set at comparable positions on the mophead. These results indicated that the mutation from the lacecap phenotype to the mophead phenotype was induced by the replacement of axillary higher order inflorescences with decorative flowers (Fig. 1). In other words, the mutation from lacecap to mophead causes the meristem identity of an axillary bud to switch from an inflorescence to a flower with large sepals and a long pedicel. Candidate genes that control both meristem identity and sepal morphology in *Arabidopsis* include *APETALA1*, a class A gene, and *SEPALLATA* class E genes [7–10]. These genes could contribute to inflorescence phenotype and sepal morphology in hydrangea. The development of DNA markers linked to the INFLORESCENCE TYPE (INF) locus has been reported. Although tightly linked INF markers have been developed, the causative gene for the mophead phenotype has not yet been identified. Waki et al. [5] constructed an SSR marker-based genetic linkage map and found that HS071 was linked to the mophead phenotype. Tränkner et al. [11] performed bulked segregant analysis using genotyping by sequencing and developed the mophead phenotype-linked markers, A109A110 and A133A134. According to the A109A110 marker-amplified sequences, two of the six alleles were linked to the recessive mophead allele. Wu et al. [12] performed a genome-wide association study (GWAS) and developed a cleaved amplified polymorphic sequence (CAPS) marker, Hy_CAPS_Inflo. Causative gene for INF should be around these markers.

The genome of *H. macrophylla* has been reported [13, 14]. One genome sequence is Hma_r1.2 sequence, which derived from ‘Aogashima-1’ introduced from its habitat in Aogashima Island [13]. The genome assembly consisted of 3779 scaffolds with a 2.2 Gb length, with a total length of 1078 Mb of 18 pseudomolecules, approximately half the length of the total genome sequence. A total of 36 930 genes were identified. Latest genome assembly is Hmc_EndlessSummerp1.0 sequence, which sequenced ‘Endless Summer’ [14]. The genome assembly consisted of 396 contigs (2.22 Gb length) that were assembled and scaffolded into the expected 18 pseudochromosomes. Utilizing genome sequencing and resequencing comparisons between alleles would contribute to clarifying the genes controlling inflorescence type and the mutations causing the mophead phenotype. Previously, *LEAFY* homologous genes and their mutations were identified as candidate causes of the double flower phenotype in *H. macrophylla* using genome sequence information and resequencing data [13]. Resequencing using short-read technology is an effective method for identifying causative mutations, particularly functional mutations caused by SNP or short INDEL. Recently developed advanced long-read technologies are powerful tools for identifying large insertions and deletions. Using long-read technologies, structural variants, such as a 10-kb retrotransposon insertion in maize and a 20-kb inverted repeat insertion in pineapple, have been detected [15, 16]. Therefore, adopting long-read technology for resequencing could effectively detect structural variant mutant alleles in hydrangea.

In this study, we aimed to identify the genes controlling the inflorescence type and the mutations causing the mophead phenotype. In this study, we identified SNPs that co-segregated with the inflorescence phenotype in a GWAS using F_2_ populations of *H. macrophylla*. The scaffold containing the *SEP* homolog locus, which was differentially expressed according to the inflorescence phenotype, coincided with the scaffold containing a marker that was strongly linked to the inflorescence phenotype. Therefore, the genome sequences of the *SEP* homolog, named *TEMARY*, were analyzed in several *H. macrophylla* cultivars. The results revealed that the *H. macrophylla* cultivars have three types of loss-of-function alleles for the *TEMARY* locus, and that the mophead cultivars carry only loss-of-function alleles.

## Results

### Prediction of the candidate gene at the INF locus

**Figure 2:**
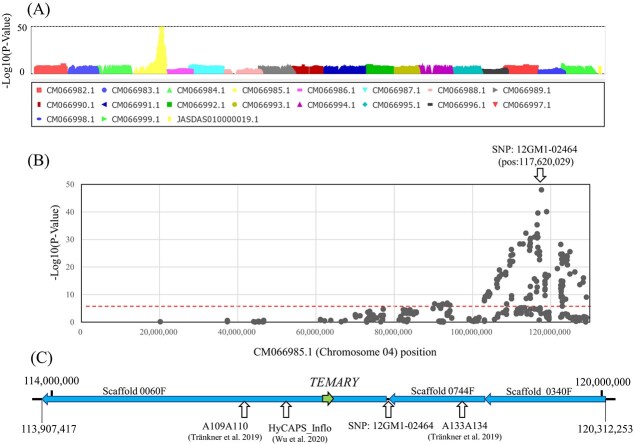
INF locus prediction using GWAS of F2 population 12GM1. (A) Manhattan plot on whole genome sequence for INF phenotype. (B) Manhattan plot on CM066985.1 (chromosome 04) sequence for INF phenotype. (C) Detailed INF locus around SNP 12GM1-02464. Nucleotide numbers correspond to Hmc_EndlessSummer_p1.0 genome. Corresponding scaffolds from Hma_r1.2 [13] are shown as blue arrows. White arrows indicate INF markers amplifying region and highest peak SNP12GM1-02464. The green arrow indicates candidate INF gene *TEMARY*.

A GWAS and a database search for previously developed INF markers were conducted to identify the INF locus in the population. For the GWAS analysis, ‘Posy Bouquet Grace’ (lacecap), ‘Blue Picotee Manaslu’ (mophead), and their F_1_ individual 12GM1 (lacecap) and selfed population of 12GM1were used. The sample included 104 lacecap and 29 mophead phenotypes, which was not deviated from the Mendelian segregation ratio of 3:1 with Chi-square test (*P* = 0.394). ddRAD-Seq data were mapped to the Hmc_EndlessSummer_p1.0 genome [14]. As a result of GWAS, 160 of 11 404 SNPs were found to be significant. All significant SNPs (−log10(*P*-value) > 5.35) were located on 90.3 to 129.1 Mb on chromosome 04 (Fig. 2A and B). The highest -log10 (*P*-value) was detected for SNP 12GM1–02464 (pos: 117620, 023 on chromosome 04) (Fig. 2B). Genomic regions supported by SNPs with more than −20 of -log10(*P*-value) values from the highest SNP are listed in Supplemental Table S1. Although no SNP completely corresponded to the genotype and phenotype, SNP 12GM1–02445, 12GM1–02463, and 12GM1–02464 showed a lower number of lacecap phenotypes with homozygous reference alleles and the highest number of mophead phenotypes with homozygous reference alleles. These SNPs supported the region around 116 703 016 to 118 954 750, which was expected to be the INF gene-locating region. Previously developed tightly linked INF markers [11, 12] were mapped from 116 278 775 to 118 423 698 bp (Fig. 2C, Supplemental Table S2). This region overlapped with the region suggested by the GWAS results.

Then, candidate causative genes of the INF phenotype in this region were searched. Because gene sequences were not provided for Hmc_EndlessSummer_p1.0 [14], the gene annotation from Hma_r1.2_1 [13] was used. Around this region, scaffolds 0060F, 0744F and 0340F from Hma_r1.2_1 genome were corresponded to 114–120 Mb region of chr04 of Hmc_EndlessSummer_p1.0 genomic sequence (Fig. 2C). In these scaffolds, 138 genes were predicted (Supplemental Table S3). To screen candidate causative gene by mRNA expression level, comparison between lacecap accessions (‘Blue Sky’ and SK-1) and their mophead sport mutant accessions (BM-1 and SKM-1, cuttings of the mutated branches) was conducted. Then, nine genes were suggested as differentially expressed (three times higher or lower in mutant accessions, FPKM in both original accessions were more than 0) (Table 1). Hma1.2p1_0060F.1_g032930 was a previously predicted candidate gene for mophead phenotype, since *Arabidopsis* homologous gene *CYP97A5* (At1g13710), which was also similar with CYP78A10 (At1g74110), was suggested as floral organ development-related gene [14, 17]. However, it did not show expression difference in mutant accessions. Among differentially expression genes, Hma1.2p1_0060F.1_g032780 and Hma1.2p1_0060F.1_g033600 were suggested as homologues of morphology related genes. Hma1.2p1_0060F.1_g033600 had similarity with *MYB106*, which was suggested as cuticular pattern formation and trichome branching in *Arabidopsis thaliana* [18, 19]. According to *Arabidopsis* homologous gene *MYB106*, this gene was suggested not to contribute shoot morphologies. In addition, Hma1.2p1_0060F.1_g033600 showed scarce expression also in original accessions. These data did not positively support the possibility that Hma1.2p1_0060F.1_g033600 is a candidate gene for inflorescence phenotype. *Hma1.2p1_0060F.1_g032780* is a partially predicted sequence of the candidate *Sepallata* sequence. This gene was predicted as candidate causative gene for the INF phenotype. As mentioned in the introduction, *SEP* is a gene that affects inflorescence structure and sepal morphology in *Arabidopsi*s, and the *SEP* homologue is a strong candidate gene for controlling inflorescence phenotype in hydrangea. Here, we name this candidate gene ‘*TEMARY*’, which means mophead phenotype in Japanese.

**Table 1:** Candidate genes for inflorescence phenotype according to RNA-Seq expression comparison between lacecap accessions and its sport mutants and previously predicted candidate gene.

			RNA-Seq expression (FPKM)			
Gene name in Hma_r1.2_1	Best hit *Arabidopsis* gene and annotation		SK-1	SKM-1	BlueSky	BM-1
Hma1.2p1_0060F.1_g033600	At3g01140	Transcription factor MYB106	0.72	0	0.43	0
Hma1.2p1_0060F.1_g033470	At3g01090	SNF1-related protein kinase catalytic subunit alpha KIN10	21.95	6.3	21.7	6.04
Hma1.2p1_0060F.1_g033330	At3g44900	Cation/H(+) antiporter 4	0.17	0	0.08	0
Hma1.2p1_0060F.1_g032830	At4g18372	Small nuclear ribonucleoprotein family protein	3.47	0	6.17	4.47
Hma1.2p1_0060F.1_g032780	At5g15800	Developmental protein SEPALLATA 1	14.22	1.6	24.29	10.99
Hma1.2p1_0340F.1_g130010	At1g74070	Peptidyl-prolyl cis-trans isomerase CYP26–2	3.39	2.38	0.69	0
Hma1.2p1_0340F.1_g130020	At1g74070	Peptidyl-prolyl cis-trans isomerase CYP26–2	1.12	0.65	0.21	0
Hma1.2p1_0340F.1_g129940	At1g49000	Transmembrane protein	2.53	3.12	0.28	0.09
Hma1.2p1_0340F.1_g129810	AT5g38690	Zinc-finger domain of monoamine-oxidase A repressor R1 protein	16.94	7.41	5.58	1.3
Hma1.2p1_0060F.1_g032930^a^	At1g74110	Cytochrome P450 family protein CYP78A10	293.52	272.72	442.38	281.04

a Suggested as candidate gene for inflorescence phenotype by Wu et al. [14].

Grey cells show three times higher or lower FPKM in sport mutants.

**Figure 3:**
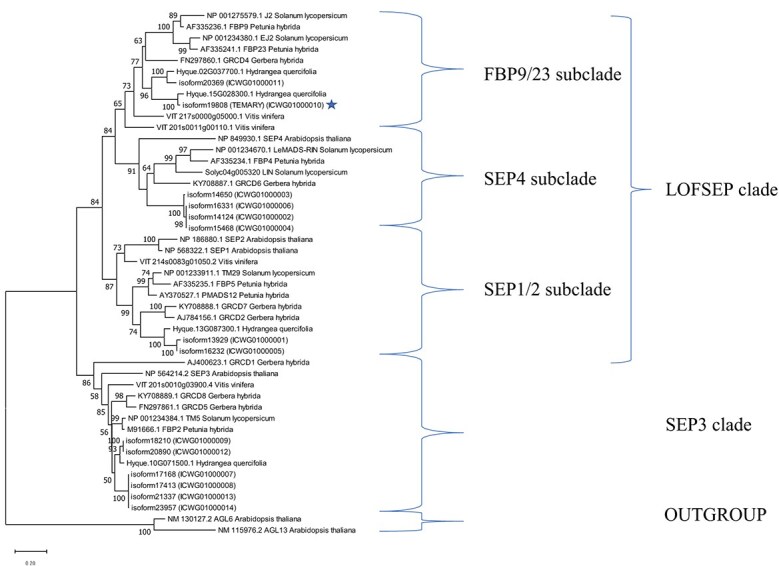
Phylogenetic analysis based on putative amino acid sequences of *SEPALLATA* (*SEP*) homologous isoforms of *H. macrophylla* and *SEP* homologs of *H. quercifolia*, *A. thaliana*, *G. hybrida*, *S. lycopersicum*, *V. vinifera*, and *P. hybrida*. There were a total of 295 positions in the final dataset. The maximum likelihood method based on the JTT model [20] with a discrete gamma distribution (+G, five categories) was applied for phylogenetic analysis using the MEGA 11 software [21]. The tree with the highest log likelihood was shown. The maximum likelihood tree had a log likelihood of −8179.06 and + G parameter of 0.7609. The tree was rooted using *AGL6* and *AGL13* as an outgroup. The bootstrap values (1000 replicates) greater than 50% are indicated at the nodes. *TEMARY* is marked with a star.

**Figure 4:**
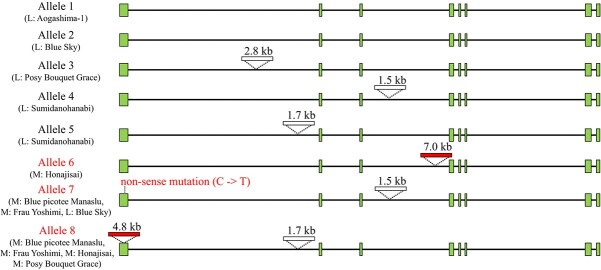
Schematic model of eight *TEMARY* alleles. Varieties that included each allele sequence in long-read sequence were shown. Insertions >1.0 kb and loss of function mutations compared to allele 1 are shown. Green boxes indicate coding sequences. The red box and red characters indicate mutations that induce functional disruption. L: lacecap variety, M: mophead variety.

### Identification and classification of *TEMARY* and *SEP* genes

*TEMARY* was suggested as partially predicted SEP family gene. To obtain full-length *TEMARY* transcript sequence and other SEP family genes, Iso-seq analysis was conducted. Then, fourteen *SEP* isoforms were identified. Fourteen *SEP* homologous isoforms were deduced to be transcripts from six loci (Supplemental Table S4 and Fig. 3). Multiple (2-4) isoforms were present at four loci. All isoforms were alleles possessed by the three samples (‘Blue Sky’, ‘Jogasaki’, Nijima-1) for Iso-Seq analysis. Of the six *SEP* homologous loci, two belonged to the SEP3 clade and four to the LOFSEP clade (Fig. 3). Among the homologous genes of the LOFSEP clade, *TEMARY* and another belonged to the FBP9/23 subclade, one belonged to the SEP1/2 subclade, and one belonged to the SEP4 subclade. *TEMARY* genomic sequence was coded on an 11 507–16 705 bp with eight exons in the hydrangea genome sequences according to mapped iso-seq sequence (Supplemental Table S5).

### *TEMARY* allelic sequence identification and comparison

**Figure 5:**
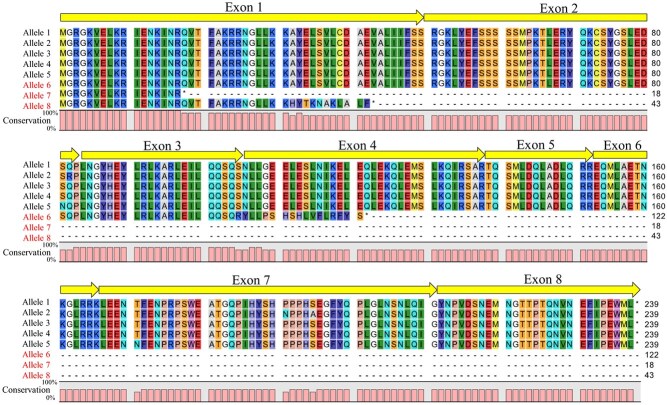
Deduced amino acid sequence alignment of *TEMARY* alleles. Alleles with red character indicate functionally disrupted alleles.

Because the lacecap phenotype is dominant, we hypothesized that *TEMARY* is functional in the dominant allele and disrupted in the recessive allele if *TEMARY* is the causative gene for the INF phenotype. Therefore, we identified and compared *TEMARY* allele sequences. Resequencing of cultivars with long-read technologies identified eight alleles (Alleles 1 to 8) with 10.4 to 17.3 kb of gene lengths (Fig. 4, Supplemental Table S6). Among them, alleles 1, 2, 3, 4, and 5 were suggested to be functional alleles because their deduced amino acid sequences produced the expected full-length protein (Fig. 5), and RNA-seq data for each allele were mapped on the expected exon region with a certain amount of mRNA expression (Supplemental Fig. S1).

**Figure 6:**
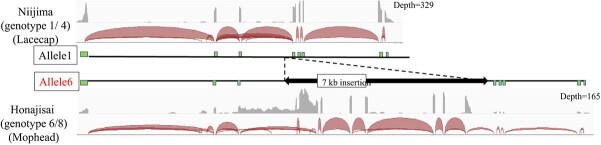
RNA-Seq mapping of *TEMARY* allele 1 in ‘Niijima’ (allele 1/4) and allele 6 in ‘Honajisai’ (allele 6/8). Green boxes indicate expected coding sequences. Gray graphs show coverage tracks, and red curves show splice junction tracks obtained by the Integrated Genome Viewer.

In contrast, three alleles (Allele 6, 7, and 8) were functionally disrupted. For allele 6, a 7-kb insertion occurred in the third intron, with abnormal splicing (Figs 4, 6, Supplemental Fig. S1). Allele 7 possessed a nonsense mutation in the first exon, resulted in premature stop codon with only 17 amino acid residues (Fig. 5, Supplemental Fig. S2). Allele 8 possessed an 4.8-kb insertion in the first exon, with very little or no *TEMARY* mRNA (Supplemental Fig. S1). According to tree clustering derived from the genomic sequence alignment from the start codon to the stop codon, allele 6 branched from allele 1, allele 7 from allele 4, and allele 8 from allele 5 (Supplemental Fig. S3). These alleles were occurred independently in their history.

In the resequenced varieties, possessing functional allele (alleles 1 to 5) led to lacecap phenotype, and possessing only disrupted alleles (alleles 6, 7, and 8) led to mophead phenotype. Haplotype genome of mophead variety ‘Endless Summer’ included alleles 7 and 8 in their haplotype sequence [14], and lacecap accessions ‘Aogashima-1’ genome sequence possessed allele 1 [13] (Supplemental Table S5), published genome sequences also showed coincidence with above rule.

**Figure 7:**
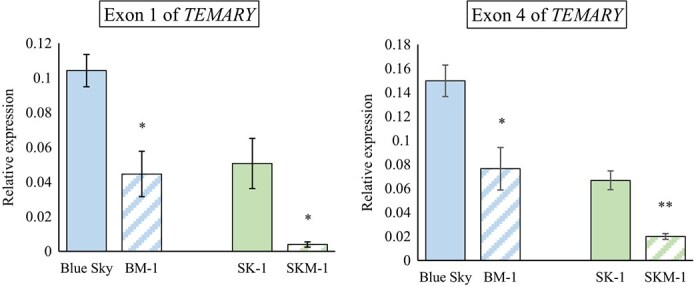
Comparison of expression levels of *TEMARY* between the original lacecap cultivars (Blue Sky, SK-1) and their sport mophead mutants (BM-1, SKM-1). Exons 1 and 4 of all *TEMARY* alleles were amplified. Inflorescence buds with a diameter of 3–5 mm were used for analysis. *HmActin* was used as an internal control. qRT-PCR analysis was performed in three biological replicates. Vertical bars indicate SE (*n* = 3). ^*^ and ^**^ indicate significant differences between the original cultivar and its sport mutant at *P* < 0.05 and 0.01, respectively, by *T*-test.

According to allelic sequences, it was clarified that ‘Blue Sky’ and BM-1 possess alleles 2 and 7, and SK-1, and SKM-1 possess alleles 2 and 8 at the *TEMARY* locus. If *TEMARY* gene is the causal gene for these sport mutants, RNA reads of functional allele 2 should be decreased or lost in sport mutants. In the mapping analysis, reduced expression levels at 0.15 to 0.18 times of the functional *TEMARY* allele 2 were observed in the sport mutants. (Table 2). In both SK-1 and SKM-1, scarce expression levels were observed on allele 8 (Supplemental Fig. S4). For ‘Blue Sky’ and BM-1, while allele 7 FPKM were similar level, allele 2 was largely decreased in BM-1 (Table 2). In BM-1, RNA mapping of exons 3 to 6 was scarcely observed (Supplemental Fig. S5). The results of expression analysis by qRT-PCR targeting all *TEMARY* alleles supported the results of the RNA-seq analysis (Fig. 7). The expression levels of the *TEMARY* gene in the sport mophead mutants were reduced compared to the original lacecap cultivars, suggesting that the difference was due to differences in the expression of allele 2.

**Table 2:** Comparison of the mRNA expression of *TEMARY* between original lacecap cultivars and their sport mophead mutants by RNA-seq analysis.

		FPKM		
Accession	Phenotype	Allele 2	Allele 7	Allele 8
Blue Sky	Lacecap	13.59	12.35	-
BM-1 (sport mutant of BlueSky)	Mophead	2.39	9.51	-
SK-1	Lacecap	17.60	-	0.11
SKM-1 (sport mutant of SK-1)	Mophead	2.72	-	0.14

### Development and application of the *TEMARY* markers

According to each allelic sequence, DNA markers for discriminating *TEMARY* alleles were developed as Temary-1, Temary-2, Temary-3, and Temary-4 (Supplemental Table S7, Supplemental Fig. S6). The combined use of Temary-2 and Temary-1 distinguished between alleles 1 and 8. According to the genotypes of the accessions, the mophead phenotype was observed when possessing only functionally disrupted alleles 6, 7, and 8, with the exception of sport mutants BM-1 and SKM-1 (Table 3). To detect causative mutations for alleles 6, 7, and 8, using agarose gel electrophoresis-based methods, Temary-3 and Temary-4 markers were additionally designed. The dCAPS marker, Temary-3, was designed to distinguish between the presence and absence of nonsense mutations in the first exon. The Temary-4 marker was designed to distinguish between the presence and absence of large insertions in the first exon. Note that the Temary-1 marker (with or without the 6-FAM label) easily distinguished between allele 6-derived PCR fragments by agarose gel electrophoresis. These markers can be used to distinguish between mutations and functional alleles.

**Table 3:** Genotypes of the *TEMARY* gene and inflorescence types in *H. macrophylla.*

Accession	Phenotype	Genotype	
Amethyst	Mophead	7	8
Benelux	Mophead	7	8
Blue Picotee Manaslu	Mophead	7	8
Bodensee	Mophead	7	8
Chibori	Mophead	8	8
Chikushi Ruby	Mophead	7	8
Chikushinomai	Mophead	7	8
Felix	Mophead	6	8
Flambeau	Mophead	6	8
Frau Mariko	Mophead	7	8
Frau Yoshiko	Mophead	8	8
Frau Yoshimi	Mophead	7	8
Green Shadow	Mophead	8	8
Honajisai	Mophead	6	8
Immaculata	Mophead	6	8
Junihitoe	Mophead	6	8
Kanuma Blue	Mophead	8	8
Madame Emile Mouillere	Mophead	8	8
Mathilde Gutges	Mophead	8	8
Mausseline	Mophead	7	8
Mis. Kumiko	Mophead	8	8
Paris	Mophead	7	8
Peach Hime	Mophead	8	8
Ruby Red	Mophead	7	8
Sensation	Mophead	8	8
Shinkai	Mophead	7	7
Uzuajisai	Mophead	6	8
03JP1	Lacecap	3	7
12GM1	Lacecap	3	8
14GT77	Lacecap	3	8
Blue Sky	Lacecap	2	7
BM-1 (mutant of Blue Sky)	Mophead	2	7
Chikushinokaze	Lacecap	3	3
Corsage	Lacecap	3	6
Dance Party	Lacecap	3	4
Fairy Eye	Lacecap	3	8
HK01	Lacecap	4	4
HK02	Lacecap	5	8
Izunohana	Lacecap	3	5
Jogasaki	Lacecap	3	8
Kirakiraboshi	Lacecap	4	4
Nijima-1	Lacecap	1	4
Posy Bouquet Kaycey	Lacecap	3	8
Posy Bouquet Grace	Lacecap	3	8
Sumidanohanabi	Lacecap	4	5
SK-1	Lacecap	2	8
SKM-1 (mutant of SK-1)	Mophead	2	8

Grey cells show mophead-linked alleles.

### Coupling of alleles between INF markers and *TEMARY*

Coupling of *TEMARY* alleles, SNP 12GM1–02464, and reported alleles of INF markers [11, 12] was performed (Supplemental Table S8). For SNP 12GM1–02464, the mophead-linked allele G was also linked to the lacecap-linked *TEMARY* alleles 1, 4, and 5. However, the genotype of the F_1_ individual 12GM1 was 3/8, which was coupled with each phenotype-linked allele in 12GM1–02464. Therefore, the phenotype and genotype were highly associated in the selfed F_2_ population. In contrast, six individuals did not cosegregate with the phenotype and genotype. Although phenotyping or handling errors were possible, unexpected cross-pollination of pollen with alleles 1, 4, and 5 of *TEMARY* was considerable.

## Discussion

### *TEMARY* as a gene responsible for the inflorescence phenotype

In expression and linkage analyses, the homeobox *SEP* homologous gene *TEMARY* was identified as a candidate gene for the mophead phenotype in *H. macrophylla*. *TEMARY* is located at the 117 Mb position on chromosome 04, where previously developed INF markers are located in close proximity [11, 12]. The presence or absence of the *TEMARY* function in each allele was coupled with a phenotype; the lacecap phenotype was observed in the presence of a functional allele, and the mophead phenotype was observed in the absence of a functional allele. These data strongly support the hypothesis that *TEMARY* is responsible for the mophead phenotype.

Recently, Wu et al. [14] suggested the possibility of *CYP78A5*, which was corresponded to Hma1.2p1_0060F.1_g032930 in Hma_r1.2 genome, as candidate gene for inflorescence shape and showed that the genotype of this gene can explain the phenotypes of the mophead cultivar ‘Endless Summer’ and the lacecap cultivar ‘Veitchii’. This gene was discovered based on its putative function in several genes located near a marker as obtained through linkage analysis. However, *TEMARY*, which is located near this gene, was not identified, even though it is the most likely candidate based on its putative function.

It was revealed that the phenotypes of 27 mophead cultivars could be explained by three types of loss-of-function *TEMARY* alleles. The pedigree in these samples were also in accordance with expected *TEMARY* genotype. ‘Chikushinomai’ was bred by Fukuoka Agriculture and Forestry Research Center, Japan (https://www.farc.pref.fukuoka.jp/hinshu/hinshu.html, In Japanese). ‘Chikushinomai’ (genotype 7/8) is the progeny of mophead accession ‘Posy Bouquet Grace’ (3/8) and lacecap 03JP1 (3/7), and 03JP1 is the progeny of ‘Jogasaki’ (3/8) and Paris (7/8). In addition to selfed population of 12GM1, we confirmed that selfed 14GT77 (3/8) (progeny of ‘Posy Bouquet Grace’ (3/8) × ‘Chibori’ (8/8)) also not deviated from the Mendelian segregation ratio of 3:1 with Chi-square test (*P* = 0.37), which included 55 lacecap and 14 mophead phenotypes. These results showed that *TEMARY* genotype was well coupled with inflorescence phenotype.

The result that the mophead phenotype can be explained by three loss-of-function alleles rather than a single allele or DNA marker strongly supports that *TEMARY* is the causal gene for the inflorescence phenotype, since the probability of this being a coincidence is much lower. Furthermore, analysis using two sets of sports mutant lines also suggested that new loss-of-function mutations, which were not present in the original cultivar, occurred in the *TEMARY* allele 2 of the sports mutants. These results suggested that *TEMARY* was responsible for the inflorescence phenotype.

### Loss-of-function *TEMARY* alleles possessed by the *H. macrophylla* cultivars

Many *H. macrophylla* cultivars have been bred by crossbreeding four mophead cultivars introduced in Europe and the USA between the latter half of the 19th century and the beginning of the 20th century. Thus, it was estimated that there were only a few types of mophead alleles in the *H. macrophylla* cultivars. In this study, three loss-of-function *TEMARY* alleles were identified that could explain the phenotypes of the mophead cultivars. Furthermore, these three loss-of-function *TEMARY* alleles coupled with previously developed INF markers co-segregated with the phenotypes of the accessions provided in each study [11, 12]. These results suggest that there are three main mophead alleles in *H. macrophylla* cultivars, all of which are loss-of-function *TEMARY* alleles. Although it has been suggested that the introduction of further alleles through recent mutations in cultivars occurs quite frequently in production areas [22]. In addition, sports mutants BM-1 and SKM-1 possibly possess new mutant alleles in allele 2. Therefore, three alleles are not all mophead alleles in the whole population. Even if new mophead genetic resources are used for breeding in the future, genetic markers for inflorescence phenotypes can be easily obtained by analyzing the sequences of their *TEMARY* genes.

Although we developed a Temary-1 marker, PCR and subsequent fragment analysis were required. Therefore, simpler and more cost-effective markers are needed for breeding programs. Three loss-of-function alleles were suggested to occur from different functional alleles (allele 6 from 1, 7 from 4, and 5 from 8) based on tree clustering of the *TEMARY* genomic sequence. Although ideal INF markers should simultaneously distinguish the three loss-of-function alleles from the functional alleles, such DNA polymorphisms are rare. We did not find such a polymorphism in genome sequence of *TEMARY*. In this respect, the Hy_CAPS_Inflo marker was suggested to be highly valuable as it could successfully distinguish phenotypes using only a single SNP.

There may be functional differences among the loss-of-function alleles. In *H. macrophylla* accessions bred in Japan before 20th century, such as ‘Honajisai’, ‘Uzuajisai’, and ‘Junihitoe’, allele 6 was included. However, recent cultivars bred in Japan rarely contain allele 6. Cultivars with genotype 6/6 were not observed. This might reflect the breeding selection history in Japan, consciously or unconsciously weeding out allele 6 according to other characteristics. Tränkner et al. [11] suggested that individuals with the B1 homozygous genotype (coupled with 7/7 in *TEMARY*) in the A109A110 marker frequently exhibited a nonflowering phenotype. Although it is not clear whether the nonflowering phenotype is caused by *TEMARY*, *TEMARY* studies may reveal relationships among *TEMARY* alleles, flowering phenotypes, and breeding selection in the future.

### Comparison of *TEMARY* between the original cultivar and its sport mutant

To confirm that *TEMARY* is the causal gene for the mophead phenotype, we used two sets of original lacecap cultivars and mophead mutants; these were ‘Blue Sky’ and BM-1, SK-1, and SKM-1. The original lacecap cultivars have heterozygous functional and disrupted alleles, and expression levels of the functional allele (allele 2) were reduced in the mophead mutants. RNA-seq mapping and qRT-PCR results suggested the expression levels of most exons in allele 2 were reduced in both mophead mutants. These suggest that in allele 2 of each mutant, a novel DNA polymorphism occurred in a region containing multiple exons or in the promoter region. In BM-1, the expression level of the functional allele 2 was decreased, while the expression level of the disrupted allele 8 was maintained at a high level, similar to that in ‘Blue Sky’. This result suggests that the decreased expression of allele 2 is not due to mutation of the upstream gene but due to mutation of allele 2. Future identification of such mutations should reveal the mechanisms for reduced expression of *TEMARY* allele 2 and would provide direct evidence for the idea that *TEMARY* gene function necessity for lacecap phenotype formation.

### The presumed function of *TEMARY* belonging to the LOFSEP clades

In the process of inflorescence development in *H. macrophylla*, the only difference between the inflorescences of lacecap and mophead is the differentiation of axillary primordia at the upper nodes near the terminal flowers in primary and axillary higher order inflorescences [2]. These primordia differentiate into axillary higher order inflorescences in the lacecap and decorative flowers in the mophead. Thus, Uemachi et al. [2] predicted that the genes that determine the inflorescence phenotype of *H. macrophylla* are likely to be involved in inflorescence determinacy. *TEMARY* is one of the *SEP* homologous genes that may be involved in inflorescence determinacy. *A. thaliana* has four *SEP* genes, of which *SEP3* plays a leading role in flower organogenesis as one of the ABCE genes [8, 23–25]. Members of the *SEPALLATA* (*SEP*) MADS-box subfamily in angiosperms are divided into two major sister clades: the SEP3 and LOFSEP clades [26, 27]. The LOFSEP clade is functionally more diverse than the SEP3 clade and its functions include controlling inflorescence determinacy and shape [28]. *TEMARY* belongs to the FBP9/23 subclade, which is one of the three subclades of the LOFSEP clade (Fig. 3). The FBP9/23 subclade includes *JOINTLESS-2* and *ENHANCER-OF-JOINTLESS-2* (*J2* and *EJ2*) of tomatoes (*Solanum lycopersicum*), which are involved in inflorescence determination [29]. *J2* and *EJ2* repress inflorescence branching; therefore, their loss-of-function mutants produce inflorescences with more flowers. The SEP gene of rice (*Oryza sativa*) *OsMADS34/PANICLE PHYTOMER2* (*PAP2*) and *SEP* homologs of *Gerbera hybrida* have similar functions to *J2* and *EJ2* of tomato [30–33]. However, the presumed function of *TEMARY* is opposite to that of these *SEP* homologous genes. The change in inflorescence structure, presumably caused by the functional deletion of *TEMARY* is the replacement of axillary higher order inflorescences with decorative flowers and the induction of inflorescences with fewer flowers [2]. In the phylogenetic tree, another *SEP* homolog in *H. macrophylla* in addition to *TEMARY* belonged to the FBP9/23 subclade (Fig. 3). Similarly, two *SEP* homologous genes in *H. quercifolia* belonged to the FBP9/23 subclade, forming a cluster with two *SEP* homologous genes in *H. macrophylla*. Gene duplication causes diversification of gene function [34]. In the genus *Hydrangea*, unique functional differentiation may have occurred after the duplication of an SEP homologous gene belonging to the FBP9/23 subclade. In the future, the involvement of *TEMARY* in inflorescence determination should be examined together with another paralog belonging to the same FBP9/23 subclade as *TEMARY* (Fig. 3). However, the loss of function of tomato *EJ2* and rice *PAP2* resulted in enlarged sepals and lemmas, respectively, which is consistent with the presumed function of *TEMARY*. This suggests that, like the LOFSEP genes in rice and tomato, *Temary* retains the ability to control the size of outer floral organs.

### Conclusion and future perspectives

The results of this study suggest that *TEMARY* is responsible for the inflorescence phenotype. In the genus *Hydrangea*, species such as *H. serrata*, *H. involucrata*, and *H. arborescens* have a clustered flowering phenotype similar to that of *H. macrophylla*. Analysis of *TEMARY* homologs will be an important approach for research on the mechanisms of inflorescence phenotypes in these species and for the development of markers. In *H. quercifolia* and *H. paniculate*, there is also a phenotype in which many decorative flowers are born in the panicle, which is an important target for breeding. The molecular mechanism underlying this phenotype is unclear. The phylogenetic tree (Fig. FPKM Accession Phenotype Allele 2 Allele 7 Allele 8 Blue Sky Lacecap 13.59 12.35 - BM-1 (sport mutant of BlueSky) Mophead 2.39 9.51 - SK-1 Lacecap 17.60 - 0.11 SKM-1 (sport mutant of SK-1) Mophead 2.72 - 0.14 ) shows that two genes belonging to the FBP9/23 subclade were present in *H. macrophylla*, and their respective orthologous genes were also present in *H. quercifolia*. This suggests that the functions of *TEMARY* are conserved in other *Hydrangea* species. The relationship between the *TEMARY* ortholog and the inflorescence phenotype of *H. quercifolia* should be investigated in the future.

## Methods

### RNA-Seq analysis and acquisition of contigs and isoforms

RNA samples from each cultivar were obtained from potted plants grown in greenhouses at the University of Shiga Prefecture, Japan. Total RNA was extracted using the RNeasy Plant Mini Kit (Qiagen, Hilden, Germany). The RNA concentration and integrity were measured using a NanoDrop spectrophotometer and an Agilent 2100 Bioanalyzer. The extracted total RNA was treated with oligo (dT) magnetic beads to isolate the mRNA, and the fragmented mRNA was used to construct cDNA. The cDNA fragments were amplified using the PCR primers. Amplified cDNA libraries were sequenced using Illumina HiSeq 2000 for paired–end–sequencing (2 × 100 bp) of SK-1 and SKM-1 samples, Illumina HiSeq 2500 for paired–end–sequencing (2 × 100 bp) of ‘Blue Sky’ and BM-1 samples, Illumina NovaSeq 6000 for paired–end–sequencing (2 × 150 bp) of ‘Honajisai’ samples, and Roche 454 GS FLX+ for single–end–sequencing (600–700 bp) of mixed RNA samples of ‘Blue Sky’ and BM-1. The reads from inflorescence buds (3–17 mm in diameter) of ‘Blue Sky’ and BM-1 using Illumina HiSeq 2500 (‘Blue Sky’: 55328676 reads, total 5588 Mbp, BM-1: 53077204 reads, total 5361 Mbp), and Roche 454 GS FLX+ (‘Blue Sky’: 55328676 reads, total 5588 Mbp, BM-1: 53077204 reads, total 5361 Mbp) were added to the RNA-Seq reads from the *H. macrophylla* leaf and flower available in the database (SRR610319: 785504 read, total 282 Mbp, SRR68791: 359683 bp, total 36 Mbp) and were assembled using a GS *de novo* Assembler version 2.6 (Roche Diagnostics) with parameters of minimum overlap length of 40 bp and minimum overlap identity of 90%.

The mixed RNA sample from inflorescence buds, leaves, decorative flowers, and nondecorative flowers of ‘Blue Sky’, ‘Jogasaki’ and a wild individual of *H. macrophylla* was reverse-transcribed using the SMARTer™ PCR cDNA Synthesis Kit (Clontech, USA). After determining the optimal number of PCR amplification cycles, cDNA was amplified using PrimeSTAR GXL DNA Polymerase (Clontech). After purification with AMPure PB Beads, the cDNA products were used to construct SMRTbell template libraries using SMRTbell Template Prep Kit 1.0 (PacBio). Sequencing was performed using the PacBio Sequel platform. Raw reads were processed into circular consensus sequence reads and subsequently clustered to consensus isoforms by Iso-Seq3 package included in PacBio SMRT Link (https://github.com/PacificBiosciences/IsoSeq).

### Phylogenetic analysis of homologous isoforms of *SEP*

Using RNA-Seq data and reads from leaves and flowers of *H. macrophylla* available in the database, 29 272 EST contigs were constructed. A BLAST search was performed on these contigs, and nine *SEP* homologous contigs were obtained (Supplemental Data S1). These contig sequences were used as query sequence for identification of the iso-seq sequence of *SEP* homologs. The *SEP* family genes of *A. thaliana*, *Vitis vinifera*, *S. lycopersicum*, *Petunia hybrida*, and *Gerbera hybrida* were used to create phylogenetic trees, referring to the work of Dreni and Ferrándiz [28] on the evolution of the *SEP* subfamily. The deduced amino acid sequences were aligned using the CLUSTAL W multiple sequence alignment algorithm, and the maximum likelihood method based on the JTT model [20] with a discrete gamma distribution (+G, five categories) was applied for phylogenetic analysis using MEGA 11 software [21]. In both phylogenetic analyses, bootstrap tests were performed with 1000 replicates.

### Prediction of the genomic locus for the mophead phenotype

To detect SNPs linked to the mophead phenotype, ddRAD-Seq analysis was performed on the 12GM1 F_2_ population [13] and their parent ‘Posy Bouquet Grace’ (lacecap), ‘Blue Picotee Manaslu’ (mophead) and their F_1_ individual 12GM1. An F_1_ individual, 12GM1, was obtained by crossing parents and then, 12GM1 was selfed to obtain F_2_ individuals. The inflorescences used for cross- or self-pollination were covered with double nonwoven fabric to prevent cross-contamination.

The low-quality ddRAD-Seq sequences were removed, and adapters were trimmed using PRINSEQ (−trim_right 1 -trim_qual_right 10 -min_len 100 -derep) and fastx_clipper (−a AGATCGGAAGAGC) in the FASTX-Toolkit (http://hannonlab.cshl.edu/fastx_toolkit; version 0.10.1). The filtered sequences were mapped onto Hmc_EndlessSummer_p1.0 [14] using Bowtie2 [35]. SNP calling was performed using BCFtools [36], and high-quality SNPs were selected by filtering conditions with a minimum read depth of 5 (−-min DP 5), a minimum SNP quality of 10 (−-minQ 10), minor allele frequency greater than 0.05 (−-maf .05), a maximum proportion of missing data of 10% (−-max-missing 0.9), and remove indels (−-remove-indels) using VCFtools [27].

GWAS analysis was performed using TASSEL 5 [38], and the GLM model was used to detect significantly associated loci. Genotypes and phenotypes were used in the GLM model, with the first five components of the PCA as the population structure matrix. The threshold values for associated SNPs were obtained by the Bonferroni correction, which was calculated as follows: −Log10(P) ≥ −Log10(0.05/11404) = 5.35.

Previously developed INF marker regions were searched in the genomic sequences of *H. macrophylla*. Each primer sequence for INF markers [5, 11, 12] was searched using BLAST.

Gene annotation of the INF locus was added using the Hma_r1.2_1 genome [13]. The corresponding gene locus for Hmc_EndlessSummer_p1.0 was searched using BLAST.

### Resequencing of *H. macrophylla* accessions and identification of the *TEMARY* allele sequence

Resequencing of *H. macrophylla* accessions ‘Honajisai’ (mophead), ‘Blue Picotee Manaslu’ (mophead), ‘Frau Yoshimi’ (mophead), ‘Blue Sky’ (lacecap), ‘Sumidanohanabi’ (lacecap), and ‘Posy Bouquet Grace’ (lacecap) was performed using a Sequel System (PacBio, Menlo Park, CA, USA). An SMRT library was constructed using the SMRTbell Express Template Prep Kit 2.0 (PacBio, Menlo Park, CA, USA) according to the manufacturer’s protocol and sequenced using SMRT Cell 8 M on a Sequel System II to obtain high-fidelity (HiFi) long reads.

Obtained sequences of each accession were converted into BLAST database using the ‘make blastdb’ function in CLC mainworkbench (Qiagen). The *TEMARY* genomic sequence in Hma_r1.2_1 [13] was used as a query, and hit sequences were extracted and aligned using the ‘create alignment’ function with the default settings, and the consensus sequence was obtained from the alignment. Raw sequence reads data of ‘Aogashima-1’ [13] was used for *TEMARY* allele sequence of ‘Aogashima-1’.

### RNA-Seq mapping *TEMARY* alleles

To compare RNA mapping pattern among alleles, RNA-Seq sequences for ‘Nijima-1’ (1/4), ‘Blue Sky’ (2/7), ‘Jogasaki’ (3/3), ‘Sumidanohanabi’ (3/3), and ‘Honajisai’ (6/8) were used. *TEMARY* alleles 1–8 were used as references. RNA-Seq data were mapped using hisat2 with default parameters and no-discordant and no-mixed options [39]. The SAM files obtained were sorted and converted into BAM files using the same tools [36]. The obtained BAM data were visualized using Integrated Genomics Viewer [40].

For comparison of *TEMARY* mRNA mapping between the original lacecap cultivars and their mophead mutant, ‘Blue Sky’, BM-1, SK-1, and SKM-1 RNA-Seq were mapped for combined sequence with Aogashima-1 genome (Hma_r1.2_1) and *TEMARY* alleles; alleles 2 and 7 for ‘Blue Sky’ and BM-1, alleles 2 and 8 for SK-1 and SKM-1. RNA-Seq mapping and visualization were performed as described above. FPKM for each gene and *TEMARY* alleles were calculated by StringTie [41].

### qRT-PCR analysis

Total RNA was reverse transcribed using ReverTra Ace (Toyobo Co., Ltd, Osaka, Japan) according to the manufacturer’s instructions. Gene expression levels were analyzed by qRT-PCR using the TB Green Pre-mix Ex- Taq II Fast qPCR (Takara-Bio, Kusatsu, Japan) with a Thermal Cycler Dice Real Time System III (Takara-Bio), according to the manufacturers’ instructions. A primer set for amplifying exon 1 was designed within exon 1 (Supplemental Table S9). For amplifying exon 4, a forward primer was designed at the boundary between exons 3 and 4, and a reverse primer was designed at the boundary between exons 4 and 5. qRT-PCR cycling was performed as follows: 95°C for 30 s, followed by 40 cycles at 95°C for 5 s and 60°C for 30 s. The comparative delta–delta Ct method was used to calculate expression levels [42]. *HmActin* was used as an internal control. qRT-PCR analysis was performed in three biological replicates.

### *TEMARY* marker development and application for accessions

Alignment of *TEMARY* alleles was performed using the CLC Main Workbench (Qiagen). DNA markers were developed based on *TEMARY* allele alignment. To discriminate between the alleles, an FAM-labeled SSR marker (Temary-1) and an INDEL marker (Temary-2) were designed. Primers were selected by visual observation. The border of the causative inserted sequences that induced functional defects was used to design primers to develop a DNA marker for detecting allele 8 (Temary-3). For the detection of nonsense mutation on allele 7, the dCAPS marker (Temary-4) was designed.

PCR amplification was performed in a 10-μl reaction mixture containing 5 μl of 2x GoTaq Master Mix (Promega), 2.5 pmol of primers, and 5 ng of template DNA for all developed markers. The DNA samples were amplified under the following cycling conditions: for Temary-1, Temary-2, and Temary-4, 35 cycles of 94°C for 1 min, 55°C for 1 min, and 72°C for 2 min, and a final extension of 5 min at 72°C. For Temary-3, 35 cycles of 94°C for 1 min, 58°C for 1 min, and 72°C for 2 min, and a final extension of 5 min at 72°C.

The PCR products were stained with 1x GRRED (Biocraft) and separated on a 2.5% (w/v) agarose gel in TAE buffer. For the Temary-4, a restriction enzyme assay was performed for the PCR product at 37°C for 3 h using NlaIII (New England Biolabs, Ipswich, MA, USA). The PCR products were stained with 1x GRRED (Biocraft) and separated on a 2.5% (w/v) agarose gel in TAE buffer. For the Temary-1 marker, the amplified PCR products were separated and detected using a PRISM 3130xl Genetic Analyzer (Applied Biosystems). The sizes of the amplified bands were scored against an internal standard DNA (400HD-ROX, Applied Biosystems, USA).

## Supplementary Material

Web_Material_uhae332

## Data Availability

Sequence reads were deposited in the Sequence Read Archive database of the DNA Data Bank of Japan (DDBJ) under accession numbers DRR510893 (transcriptome reads for a mixture of Blue Sky and BM-1) and DRR510894–DRR510900 (genome reads for the seven lines). *TEMARY* alleles 1 to 8 sequences were registered under the accession numbers LC795557 to LC795564 in DDBJ. Transcriptome reads for ‘Niijima-1’, ‘Blue Sky’, ‘Jogasaki’, ‘Sumidanohanabi’, and ‘Honajisai’ have been reported in Nashima et al. [13], under the accession number DRA010300.
