# Supplementary figures and images for: The SEP homologous gene TEMARY regulates inflorescence phenotypes in Hydrangea Macrophylla
Source: Hortic Res. 2024 Nov 26;12(3):uhae332. doi: 10.1093/hr/uhae332 (PMC11879557; doi:10.1093/hr/uhae332)

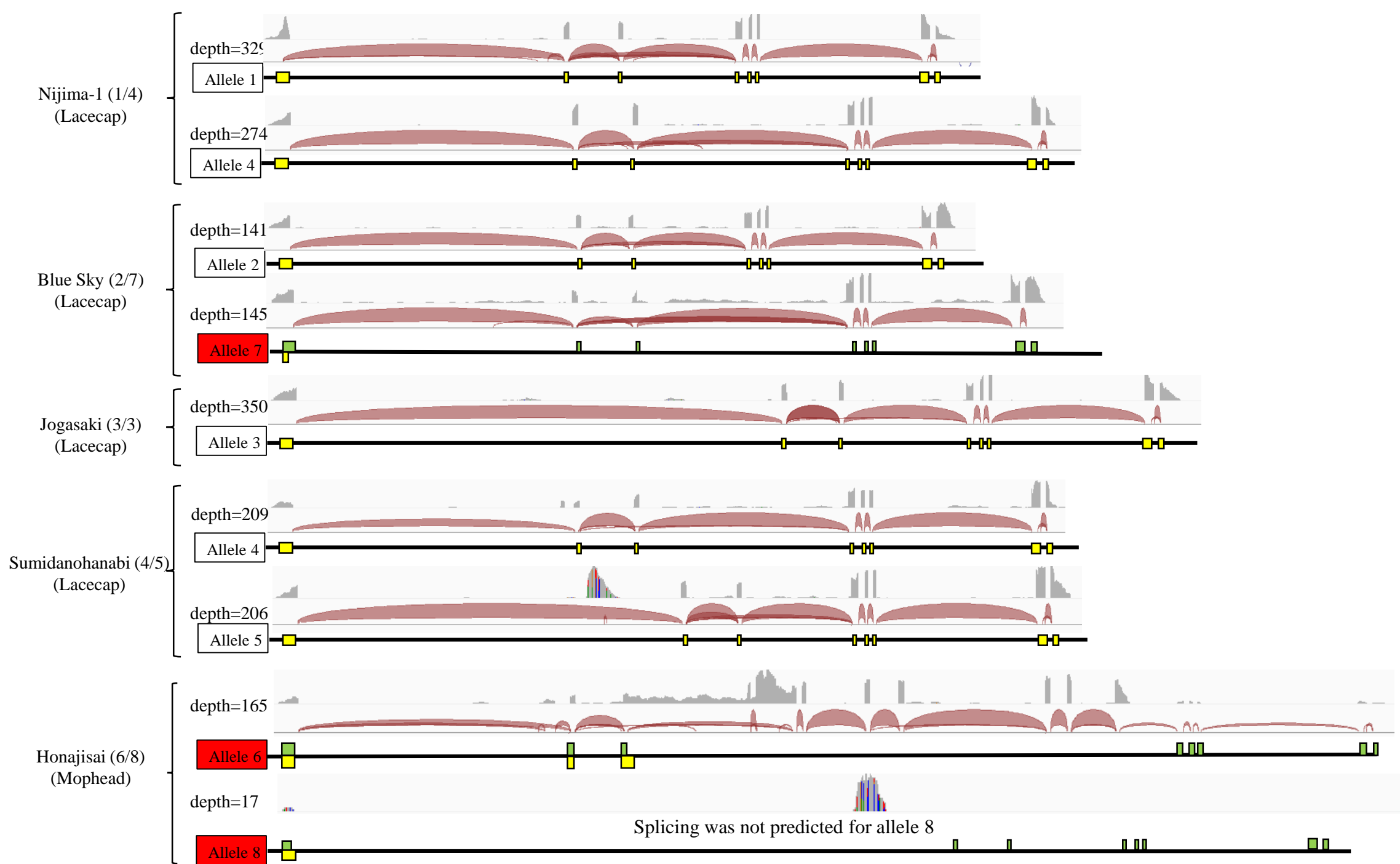

Supplement: Web_Material_uhae332 [file web_material_uhae332.zip › Supplemental Fig. 1.pdf]

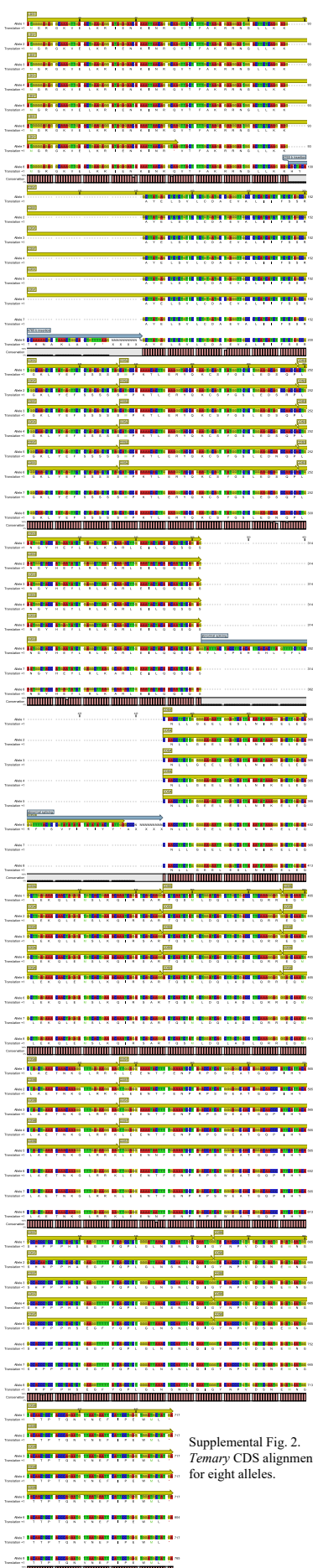

Supplement: Web_Material_uhae332 [file web_material_uhae332.zip › Supplemental Fig. 2.pdf]

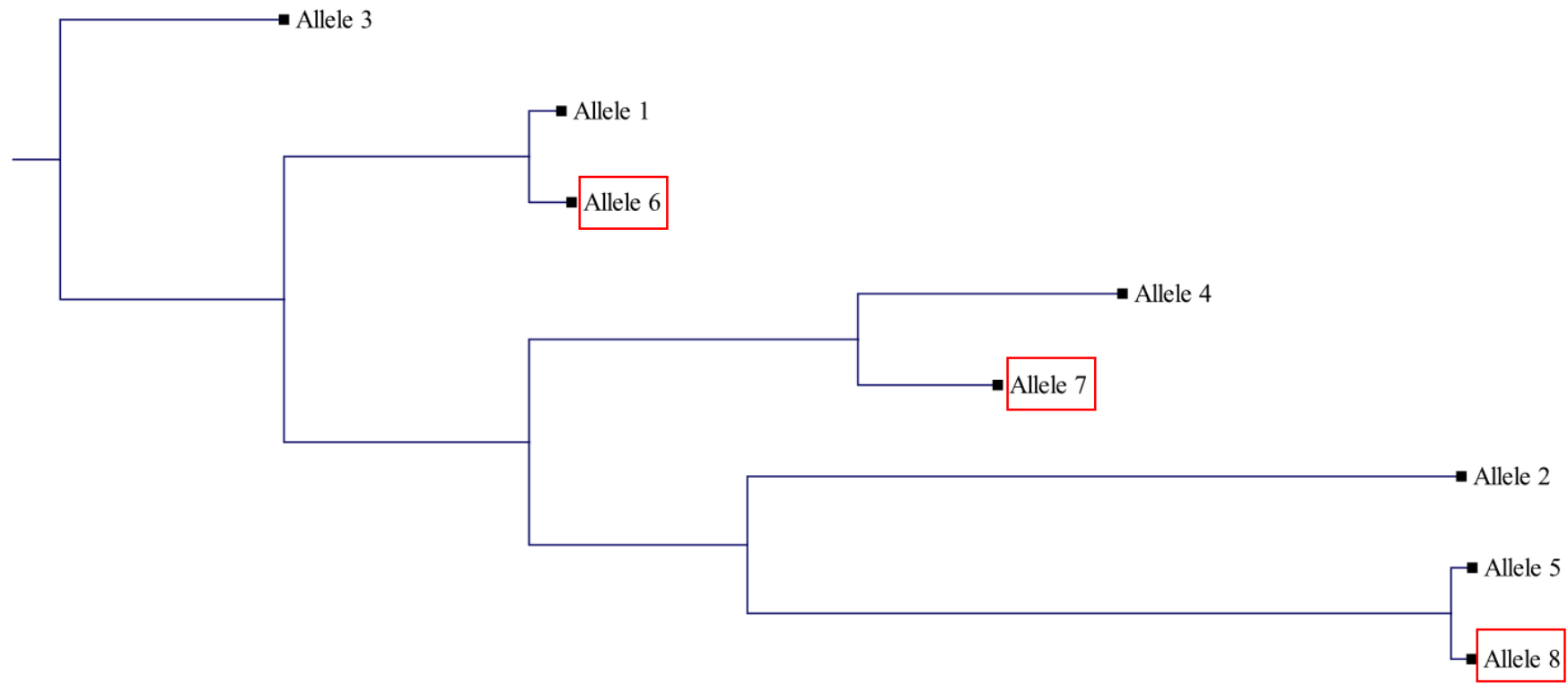

0.013

Supplement: Web_Material_uhae332 [file web_material_uhae332.zip › Supplemental Fig. 3.pdf]

Allele 2

Allele 8

SK-1

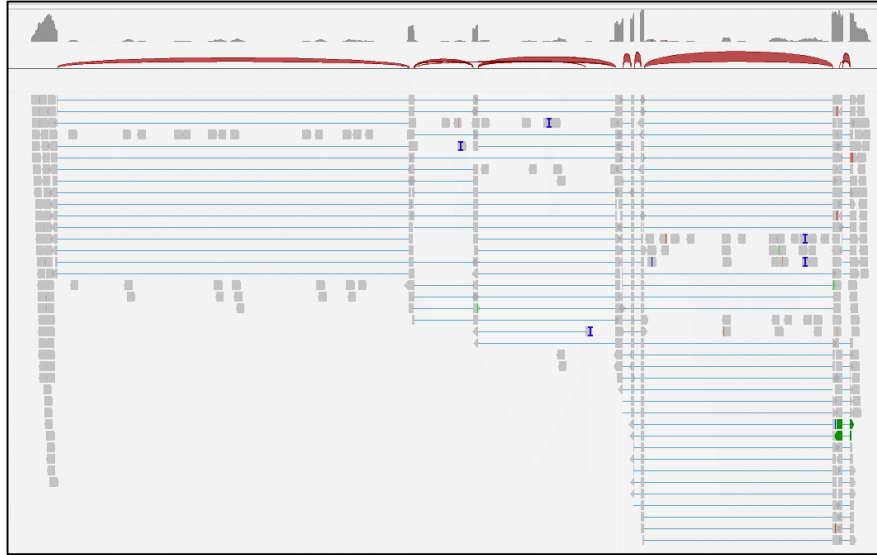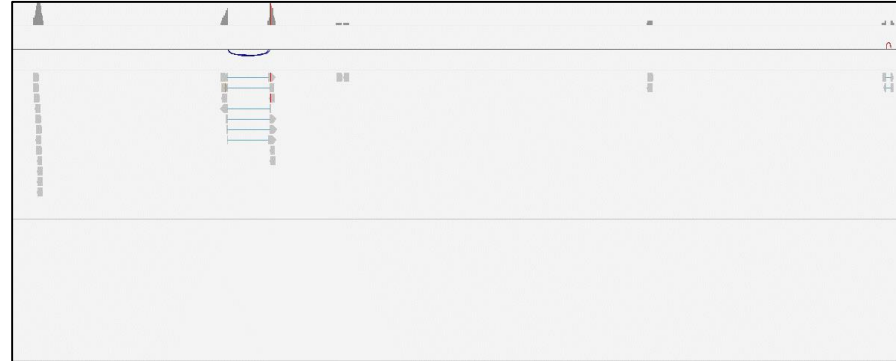

SKM-1

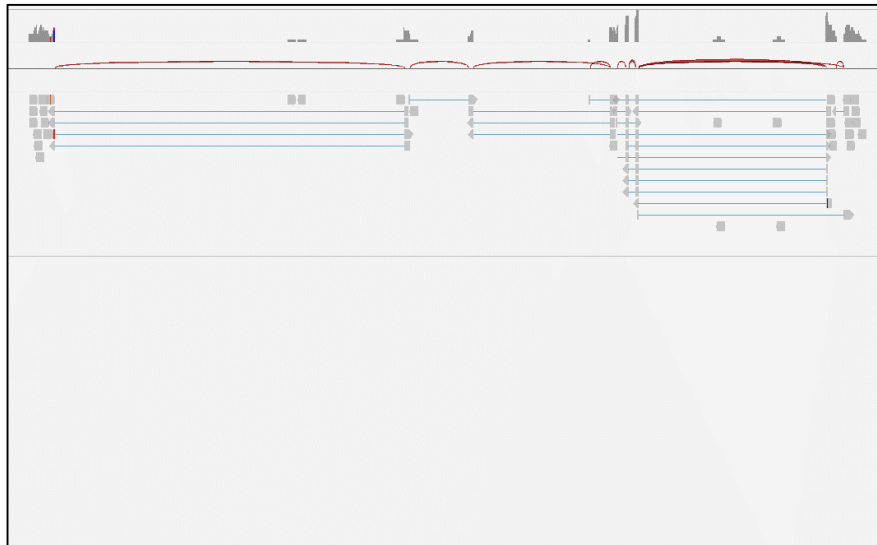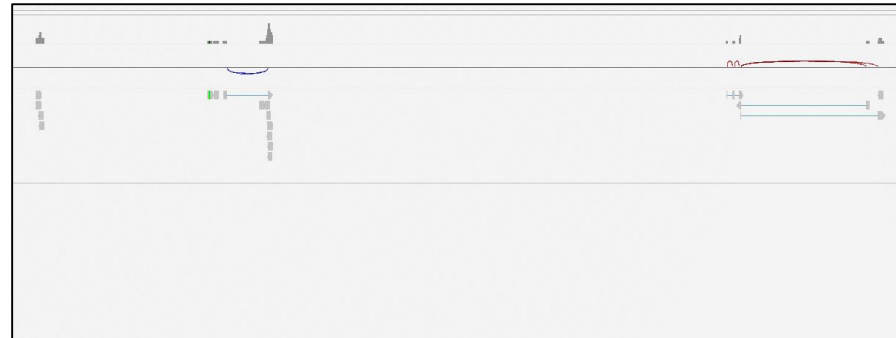

Supplement: Web_Material_uhae332 [file web_material_uhae332.zip › Supplemental Fig. 4.pdf]

Allele 2

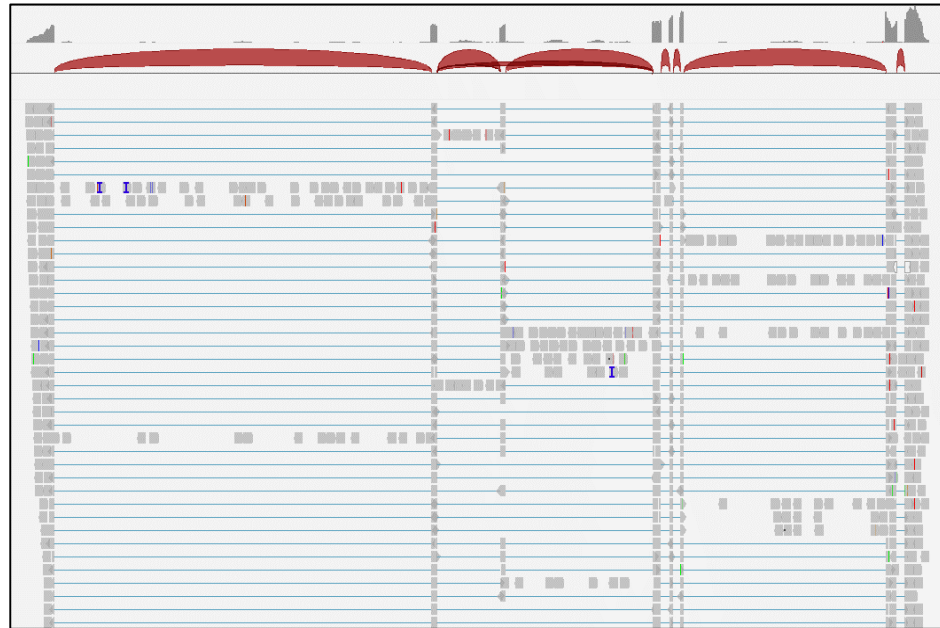

Allele 7

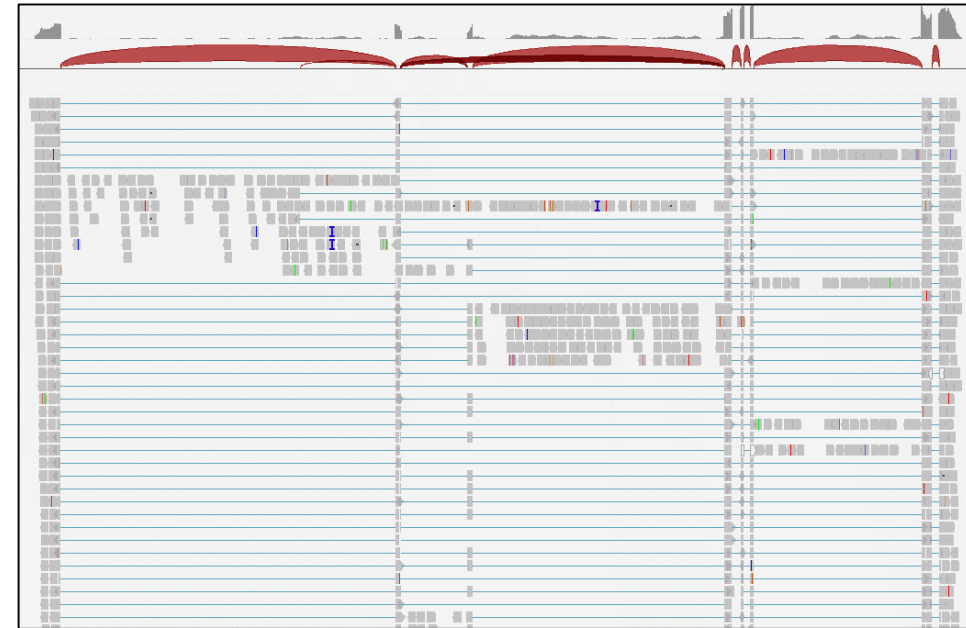

Blue Sky

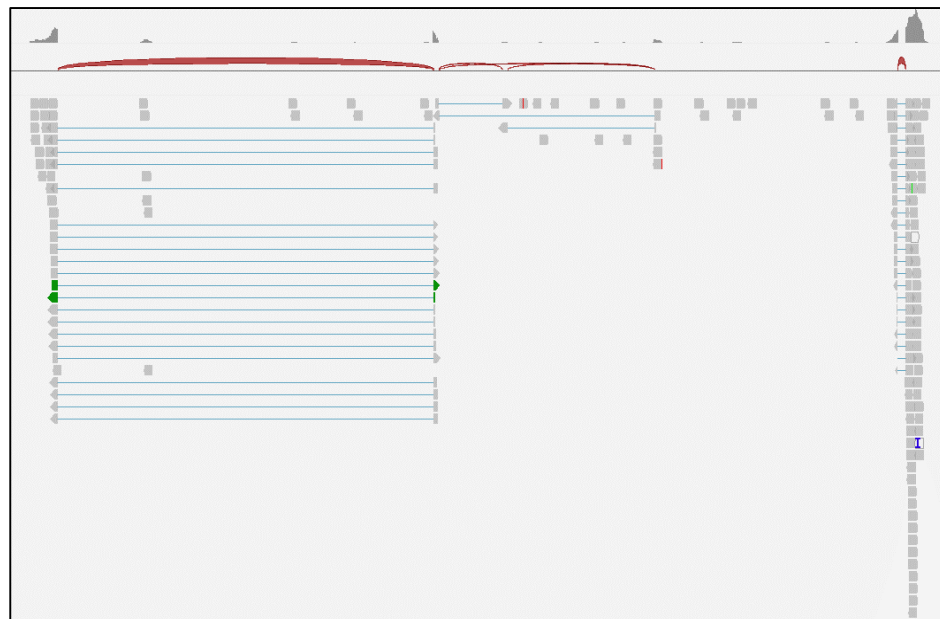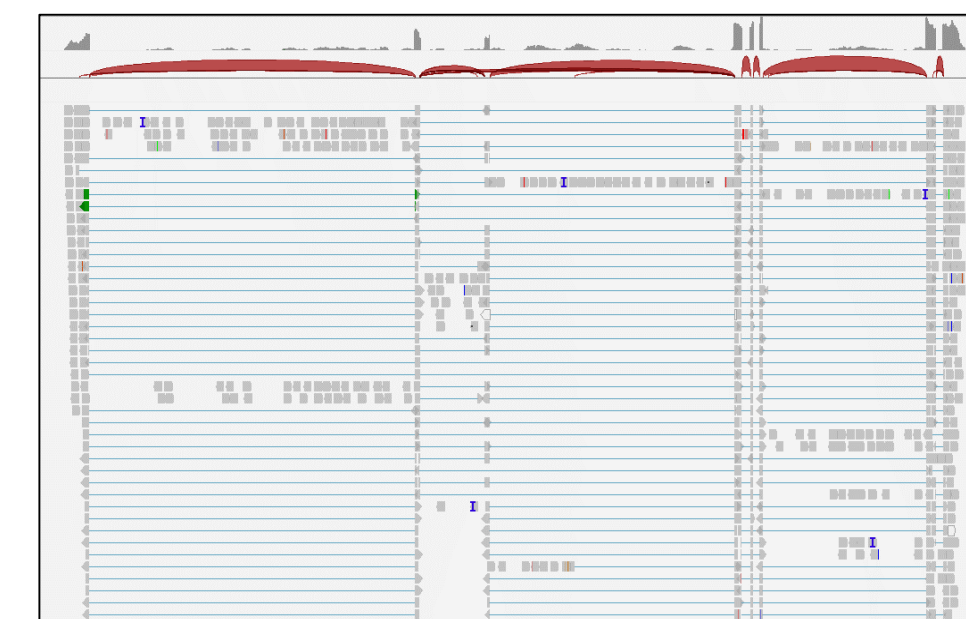

BM-1

Supplement: Web_Material_uhae332 [file web_material_uhae332.zip › Supplemental Fig. 5.pdf]

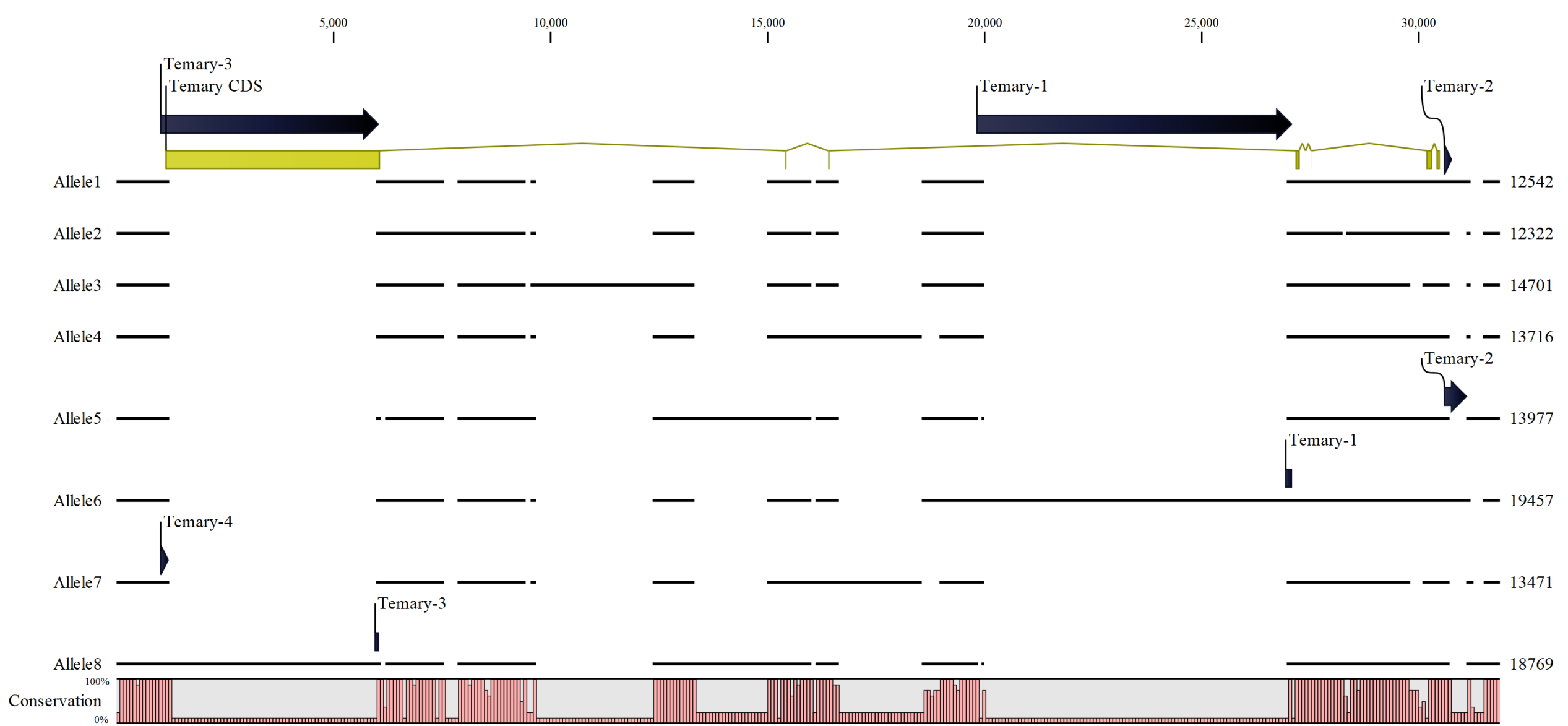

Supplement: Web_Material_uhae332 [file web_material_uhae332.zip › Supplemental Fig. 6.pdf]
